# High-Throughput Sequence Typing Reveals Genetic Differentiation and Host Specialization among Populations of the *Borrelia burgdorferi* Species Complex that Infect Rodents

**DOI:** 10.1371/journal.pone.0088581

**Published:** 2014-02-12

**Authors:** Maude Jacquot, Maxime Bisseux, David Abrial, Maud Marsot, Elisabeth Ferquel, Jean-Louis Chapuis, Gwenaël Vourc'h, Xavier Bailly

**Affiliations:** 1 INRA, UR346 Épidémiologie Animale, Saint Genès Champanelle, France; 2 MNHN-CNRS-P6, UMR 7204 Conservation des Espèces Restauration et Suivi des Populations, Paris, France; 3 Institut Pasteur, CNR Borrelia, Paris, France; University of Kentucky College of Medicine, United States of America

## Abstract

Lyme disease is a zoonosis caused by various species belonging to the *Borrelia burgdorferi* bacterial species complex. These pathogens are transmitted by ticks and infect multiple, taxonomically distinct, host species. From an epidemiological perspective, it is important to determine whether genetic variants within the species complex are able to spread freely through the whole host community or, instead, if certain variants are restricted to particular hosts. To this end, we characterized the genotypes of members of the *B. burgdorferi* species complex; the bacteria were isolated from more than two hundred individuals captured in the wild and belonging to three different rodent host species. For each individual, we used a high-throughput approach to amplify and sequence *rplB*, a housekeeping gene, and *ospC*, which is involved in infection. This approach allowed us to evaluate the genetic diversity both within and among species in the *B. burgdorferi* species complex. Strong evidence of genetic differentiation among host species was revealed by both genes, even though they are, *a priori*, not constrained by the same selective pressures. These data are discussed in the context of the advancements made possible by multi-locus high-throughput sequencing and current knowledge of Lyme disease epidemiology.

## Introduction

A large percentage of the pathogens that infect humans are zoonotic [1] and are maintained in local host reservoirs. Pathogens can be specific to a single host species, or they can possess a more generalist transmission strategy and infect multiple, unrelated host species [2]. Identifying these hosts remains a challenging task that can have crucial implications for infectious disease management strategies [3].

Studying the genetic diversity of zoonotic pathogens can provide valuable insights into various aspects of their epidemiology. For example, analyses of the diversity of pathogens within infected individuals can reveal information about the exposure and/or the susceptibility of hosts to different pathogen genotypes. Furthermore, cases of host co-infection, in which a host is simultaneously infected by different pathogen genotypes, are influenced by the contact dynamics between hosts and infectious agents, the establishment of host defenses, and potential interactions between and among pathogen genotypes. Finally, observations of genetic differentiation between pathogens that infect different host species provide information about genetic exchanges between pathogens found in different potential reservoirs and potential specialization events.

With these goals in mind, the *Borrelia burgdorferi* species complex is an ideal model system in which to study the spread of zoonotic pathogens within a community of hosts. This species complex includes the pathogens that cause Lyme borreliosis, one of the most frequent vector-borne zoonotic diseases in the Northern Hemisphere. Bacteria of the *B. burgdorferi* species complex are acquired and transmitted by ticks during their blood meals and infect many different host species, including small mammals, birds, and reptiles. While species of the complex are thought to have host ranges restricted to one or a few host species, the host specificity of individual bacterial genotypes has yet to be evaluated [4]. Finally, this species complex also provides an opportunity to study co-infection, as individual hosts are usually infested by several ticks, each of which can transmit a different bacterial genotype [5].

The process of infection by members of the *B. burgdorferi* species complex involves many different bacterial genes. Among these, the outer surface protein encoded by the *ospC* gene is characterized by a peculiar pattern of polymorphism, one that is shaped by diversifying selective pressures that arise as a function of host diversity at both a genetic and an immunological scale. In addition, recombination plays an important role in the evolution of this genome region [6], and the shuffling of genotypes caused by recombination could result in the disruption of any potential associations between *ospC* genotypes and those of housekeeping genes. However, residual association might still result from either selective or epidemiological constraints that induce linkage disequilibrium at the whole genome scale. Therefore, by comparing the patterns of diversity found in housekeeping genes with those found in infection-related genes, we can obtain information about the evolution of the *B. burgdorferi* species complex.

Recent developments in sequencing technology have provided the opportunity to investigate the genetic diversity of pathogens within and among hosts in greater detail. Massive parallel sequencing can be used to obtain numerous sequences from the pathogens within a single host and to characterize multiple loci within each pathogen. Moreover, pathogens infecting several hosts can be characterized in a single high-throughput sequencing run, providing an efficient way to perform studies across a community of hosts.

In this context, the aim of this study was to describe patterns of genetic differentiation within and among populations of the *B. burgdorferi* species complex that infect three rodent species. Hosts were sampled in a French peri-urban forest over the course of several years, and bacteria were characterized using High-Throughput Multi-Locus Sequence Typing (HiMLST) [7]. This aimed at obtaining an accurate description of genotypes that infect the studied hosts. Characterization of pathogens targeted both a housekeeping gene and an infection-related gene, differentiated by different phylogenies, to evaluate potential evolutionary scenarios. Results are discussed in the context of current knowledge of molecular epidemiology into the *B. burgdorferi* species complex.

## Materials and Methods

### Ethic statement

All conducted experiments complied with the current laws of France. Trapping and collection of rodents conducted on the study site (Forêt de Sénart, Essonne, France) were carried out under the control of Laurent TILLON (Office National des Forêts), Head of Research Group mammals. The project was approved by the Ethics Committee in Animal Experiment (CEMEA Auvergne). Rodents were euthanized by cervical dislocation. Ear punch biospsies were limited to the minimum size needed, the puncture was disinfected with hydrogen peroxide and checked before releasing the animal.

### Sample selection

As part of another study, Siberian chipmunks (*Tamias sibiricus barberi*), bank voles (*Myodes glareolus*), and wood mice (*Apodemus sylvaticus*) were caught during field sampling performed in the forest of Sénart (3200 ha, 48°40′N, 02°29′E), located near Paris (France), over the course of six years, from 2005 to 2010. An ear biopsy was taken from each animal, from which we extracted DNA; PCR Restriction Fragment Length Polymorphism (RFLP) analysis was then used to test whether the animals were infected with bacteria belonging to the *B. burgdorferi* species complex [8] [9].

As the aim of this study was to evaluate, in depth, the genotypic diversity present within these hosts, we selected 228 infected individuals (Table S1) using a random stratified sampling strategy, targeting a maximum of 30 individuals per host species per year, as long as there were enough samples; the sample contained 125 chipmunks, 93 bank voles, and 10 wood mice.

### Sample characterization

The purpose of this study was to compare the patterns of genetic differentiation observed in a housekeeping gene with those from a gene obviously affected by host-driven selective pressures. From among the different markers included in the MLST scheme that has been developed for the *B. burgdorferi* species complex, we focused on partial sequences of *rplB*, a housekeeping gene. We also studied partial sequences of *ospC*, an infection-related gene with antigenic properties that is affected by balancing and/or negative frequency-dependent selective pressures that arise from host-pathogen interactions [10] [5]. Evidence of these evolutionary patterns can be observed in *ospC* sequence data and seem to be widespread among species within the *B. burgdorferi* species complex.

Markers were amplified independently via semi-nested PCR for each DNA sample using the GoTaq kit (Promega, Fitchburg, USA). Outer amplifications were performed using the modified primers rplB_Out_F (5′ AGGGTATTAAGACTTATAAGC 3′) and rplB_Out_R (5′ AGGCTGTCCCCAAGGAGAYAC 3′) for *rplB* [11]. For *ospC*, primers ospC_F1 (5′ GGGAWCCAAAATCTAATAYAA 3′) and ospC_R1 (5′ ATATTGACTTTATTTTTCCAGTTAC 3′) were used [6]. Amplifications were performed using a total volume of 25 µl, which included 8 µl of DNA extract, 0.25 µl of Taq polymerase, 10 pmol of each primer, and the buffer provided by the manufacturer at a final concentration of 1X. Amplifications were performed using the following reaction cycle parameters: an initial denaturation step at 95°C for 10 min, followed by 40 cycles, each composed of a denaturation step at 94°C for 30 s, an annealing step of 30 s (at 58°C for *rplB* or 56°C for *ospC*), and an elongation step at 72°C for 40 s. These cycles were followed by a final elongation step of 5 min at 72°C.

For the inner PCR amplifications, the forward primers for each region were composed of two parts: 1) a 30-base-pair (bp) oligonucleotide required for the unidirectional sequencing process on a 454 platform (5′ CCTATCCCCTGTGTGCCTTGGCAGTCTCAG 3′) and 2) a gene-specific oligonucleotide to bind the sequence region of interest. The target-specific oligonucleotides were the primers rplB_In_F (5′ CGCTATAAGACGACTTTATC 3′) for *rplB* [11] and ospC_F2 (5′ AAAAGGAGGCACAAATTAATG 3′) for *ospC* [12]. Similarly, the reverse primer was composed of three parts: 1) a 30-bp oligonucleotide required for sequencing on a 454 platform (5′ CCATCTCATCCCTGCGTGTCTCCGACTCAG 3′); 2) a 12-bp oligonucleotide tag corresponding to the individual host from which the DNA had been extracted; and 3) rplB_Out_R or ospC_R1, which had already been used for the outer PCR amplifications. Inner amplifications were performed using the same thermal cycling parameters as described above, except that the annealing temperature was fixed at 58°C for both sets of primers.

For each locus, 10 µl of the PCR product obtained from the inner amplification reaction was mixed in a 15-ml tube (BD Biosciences, San Jose, USA). The mixture was vortexed, and 2 ml were pipetted and purified using the NucleoSpin PCR Clean-up Kit (Macherey-Nagel, Düren, Germany). The quantity of DNA after purification was assessed by spectrophotometry at 260 and 280 nm (Nanodrop, Thermo Scientific, Wilmington, USA). Equal amounts of purified *rplB* and *ospC* PCR products were then mixed with each other and sent to GATC-Biotech (Konstanz, Germany) for sequencing on 1/16th of a GS FLX+ run.

### Sequence analysis

#### Assignment of sequences to individuals and loci

Raw sequences were assigned to their respective individual host animals and target loci using a Blast approach [13]. We matched the individual-host-specific 12-bp tag at the beginning of each sequence with our reference tag sequences using BlastN. To ensure the best possible assignment for each sequence, Blast searches were performed using the following parameters: the query strands to search against database for BlastN was fixed at 1; the reward for nucleotide match was fixed at 4; the penalty for a nucleotide mismatch was fixed at 5; the gap opening cost was fixed at 3; the gap extending cost was fixed at 5; and the used word size was fixed at 4. The best hit was saved and used to assign each sequence to an individual host.

To assign each sequence to its respective locus (*rplB* or *ospC*), we then used BlastN to compare each sequence to those in a database that contained different representative sequences of the target loci from the *B. burgdorferi* MLST database (http://borrelia.mlst.net) [11] and from GenBank [14]. Blast searches were performed using the default parameters. Sequences were assigned to a given locus based on the best Blast hit if the length of the hit was longer than 130 bp with a percentage of identity higher than 80%. Nucleotides corresponding to primers were removed, and assigned sequences were saved for further analysis.

#### Trimming of the sequence dataset

After checking the distribution of mismatches along the alignments of the assigned sequences, we decided to use only the first 500 bp of the sequences for subsequent analyses (data not shown). We also removed from the dataset all sequences shorter than 350 bp, of which there were relatively few. We constructed two alignments of the respective sequences of each locus of each individual using K-Align [15].

#### Genotype delineation

A major drawback of high-throughput sequencing methods is the relatively high base-calling error rate. This issue could present a problem for within-species studies of diversity as analyses are likely to be affected by the introduction of low-frequency variants that are, in reality, artifacts of sequencing error. In order to circumvent this problem, genotypes and genotype groups were delineated according to two successive distance-based nearest-neighbor classifications.

First, we used the sequence alignments obtained for each locus from each host individual to define a subset of nucleotide sites that contained only sites with Single-Nucleotide Polymorphisms (SNPs); in order to be included in the subset, each site had to be successfully base-called in more than 40% of sequences and had to contain at least two alleles that were present in more than 5% of the sequences in the alignment. We then computed pairwise distances between the sequences contained in each alignment based on the number of selected SNPs in which they differed. Gapped positions were ignored during pairwise comparisons. Finally, sequences were clustered so that the pairwise distance between a given sequence and another of the same group (i.e. genotype) was equal to or lower than a locus-specific threshold. These thresholds were fixed according to the diversity pattern of each marker. As *rplB* appeared to contain little intra-specific diversity, we set the threshold for divergence at one nucleotide site. However, we observed more genetic divergence within the *ospC* sequences and therefore set the *ospC* threshold at two sites. For all of the genotypes identified, we then obtained consensus sequences based on the majority rule for each locus in each individual host.

Next, to identify genotypes that were present in different host individuals, all consensus sequences and singletons obtained from our intra-individual analysis were grouped together according to the algorithm described above. Then, a majority-rule consensus sequence was found for each genotype that was shared across multiple hosts. To ensure robust data, genotypes that were represented by three or fewer sequences in the larger dataset were removed before subsequent analyses.

#### Differentiation among host species and sampling years

In order to assess the influence of various structuring factors on the observed genetic diversity, we performed an analysis of molecular variance (AMOVA) on the sequence dataset [16], with host species, sampling year, and host individual as structuring factors. This analysis was based on the genotypes defined above, and, within individual hosts, each genotype was weighted according to the number of sequences it contained. In order to prevent bias in the analysis resulting from the impact of inter-specific divergence among Borrelia species, genotypes were considered to be genetically equidistant from each other. The analysis was performed with the amova function in the ade4 library in R [17].

#### Genotype phylogeny and delineation of genotype groups

We used a phylogenetic approach to further examine the diversity of the observed genotypes. For each locus, consensus sequences and reference sequences were aligned to each other using K-Align [15] these reference sequences were the same as those that had been previously used for locus assignments. Phylogenetic searches were performed with a maximum likelihood approach using PhyML [18],and we chose the most appropriate model of evolution for each alignment according to the Akaike Information Criterion (AIC) [19] calculated using the APE library in R [20]. A phylogenetic network was obtained for each locus using the Neighbor-Net method [21] in SplitsTree4 [22]. The distance matrices used to create the networks were computed from alignments with Paup* 4.0 b10 [23] using the best model for each locus, i.e. GTR+G for rplB and GTR+I+G for ospC [24] [25]. The substitution rate matrices were estimated via maximum likelihood and assuming empirical nucleotide frequencies. As calculated by the PhyML analysis, the shape parameter of the gamma distribution for rplB was fixed at 0.227; for ospC, the proportion of invariable sites and the shape parameter of the gamma distribution were fixed at 1.117 and 0.169, respectively.

Using the networks, we were able to empirically delineate groups of closely related genotypes. We were then able to calculate the proportion of individuals within each host species that was infected by any given group of closely related genotypes.

#### Genotype associations and co-occurrence

To search for multi-locus associations, i.e. associations between the different genotypes of the two markers, a graph analysis illustrating the frequency of co-occurrence of the different genotype groups within hosts was performed for both marker. First, we constructed an incidence matrix (m*n), with m as the number of individual hosts and n as the number of genotype groups. This matrix described the presence or absence of the different genotype groups among host individuals. We then created a co-occurrence matrix (n*n) that described the amount of co-occurrence of genotype groups within individuals by multiplying the incidence matrix by its transpose. One-half of the resulting matrix provided the information required to build a graph that described the co-occurrence of genotypes over all individual hosts. Second, we evaluated preferential associations among genotype groups using a “greedy” approach [26] to assess modularity. Measures of modularity aim to determine the adequacy of different classification schemes in representing clusters and divisions in datasets; here, the clusters represented the co-occurrence of genotypes in individual hosts. In the first step of this analysis, each genotype group was considered to be a set. At each step, the algorithm combined two sets such that the resulting classification had the highest degree of modularity as compared to all possible combinations of sets; this was performed until a single set remained. We calculated estimates of modularity for each classification by comparing the fraction of co-occurrence of genotype groups that fall within the sets of the classification with the expected value of the same quantity if the co-occurrences happened randomly without regard for sets of classification [27]. In the final step of the analysis, the classification with the highest modularity from all the generated classifications was selected.

This analysis was performed using the igraph library in R [28]. The co-occurrence matrix was obtained from the incidence matrix using the graph.incidence and the bipartite.projection functions. The classification analysis was performed using the fastgreedy.community function, and graphical output was produced using the tkplot function.

Finally, to obtain quantitative information on the frequency of co-infection by the different genotypes identified in the host community, we used the data from each marker to calculate how frequently an individual host was infected by at least two different genotypes.

## Results

In total, we obtained 16,913 sequences. Of these, 16765 of them were assigned to individual hosts and deposited to the Sequence Read Archive (SRA) database under the accession SRP032755.

16,222 sequences, i.e. 95.9% of the total raw sequences, were assigned to individual hosts and one of the two loci. In the sample of all of the 228 studied mammals, we obtained, on average, 33.2 *rplB* sequences and 37.9 *ospC* sequences per host individual. A few individuals did not yield any sequences, but we were able to successfully obtain *rplB* sequences from 96 chipmunks, 92 bank voles, and 8 wood mice, and *ospC* sequences from 94 chipmunks, 90 bank voles, and 5 wood mice.

**Figure 1:**
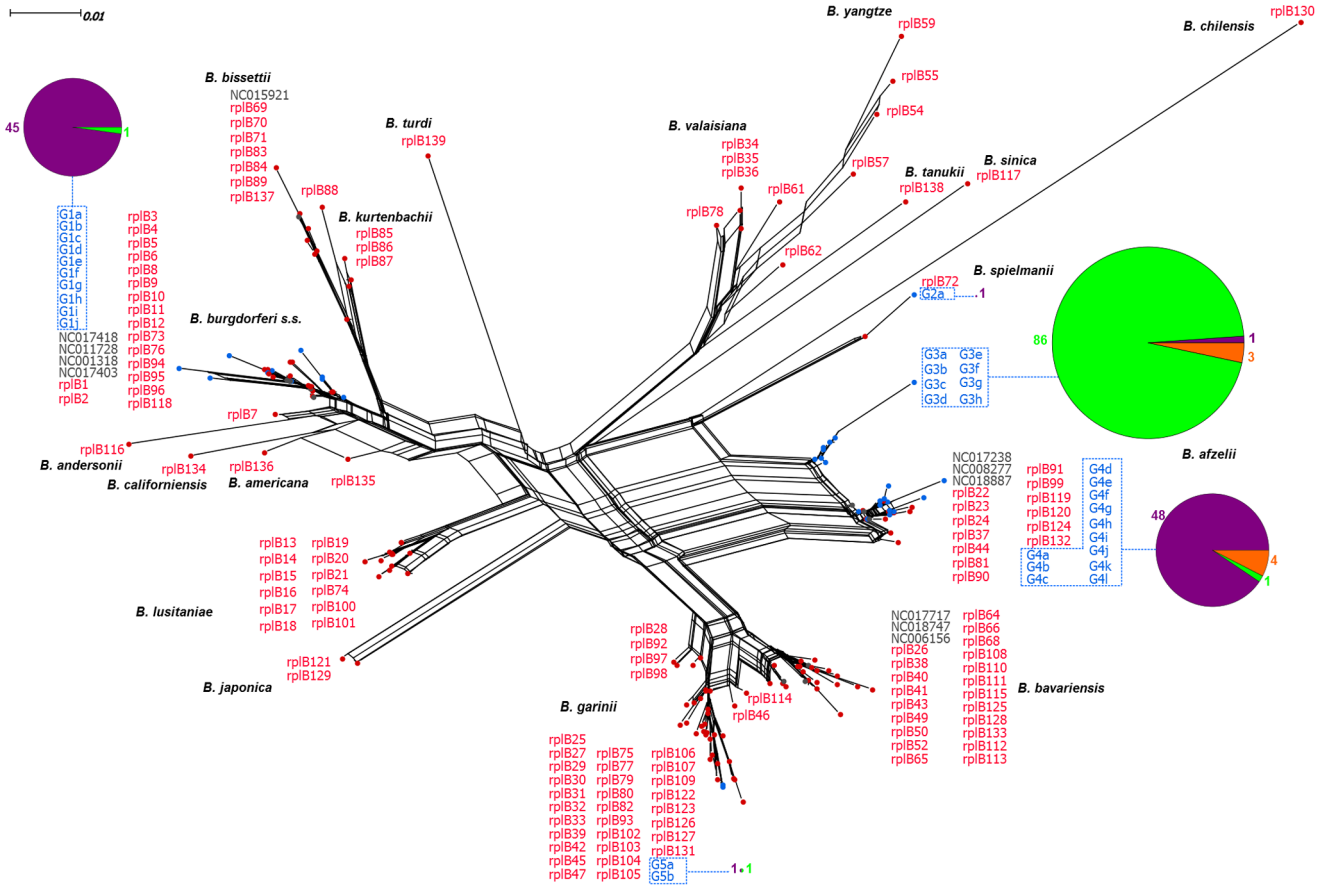
Phylogenetic network of *rplB* sequences. The network includes consensus sequences of the genotypes recovered in this study (blue) as well as reference sequences for species in the *Borrelia burgdorferi* complex. The genotypes identified in this study formed empirically delineated genotype groups that are indicated with blue dotted lines. The pie charts connected to each main genotype group show the proportion of genotypes in the group that were isolated from each host species: Siberian chipmunks (purple), bank voles (green), or wood mice (orange).

Using the nearest-neighbor classification algorithm, genotypes were delineated from the sequences for each locus; genotypes containing fewer than four sequences were excluded from subsequent analyses. Consequently, our *rplB* dataset included 33 unique genotypes identified from 87 chipmunks, 87 bank voles, and 7 wood mice. In the phylogenetic analysis based on the *rplB* data, these genotypes formed five major genotype groups (Figure 1). Most of these groups, with the exception of the group G3, were closely related to previously described, reference sequences. Genotype groups G3 and G4, whose members had been recovered from 86 bank voles and 48 chipmunks, respectively, clustered with sequences of *B. afzelii*. Sequences of the G1 genotype group were isolated from 46 chipmunks; the sequences seemed to correspond to those from *B. burgdorferi*, while G2 genotype sequences grouped with the sequence of the *B. spielmanii* rplB72 allele. Genotype group G5, whose sequences clustered with those of *B. garinii*, was recovered from only two individual hosts. Within each genotype group, most sequences were obtained from only a single host species, as most members of the G1, G2, and G4 genotypes were isolated from chipmunks and the majority of the G3 sequences came from bank voles. The relatively few sequences recovered from wood mice were found in groups G2 and G4.

**Figure 2:**
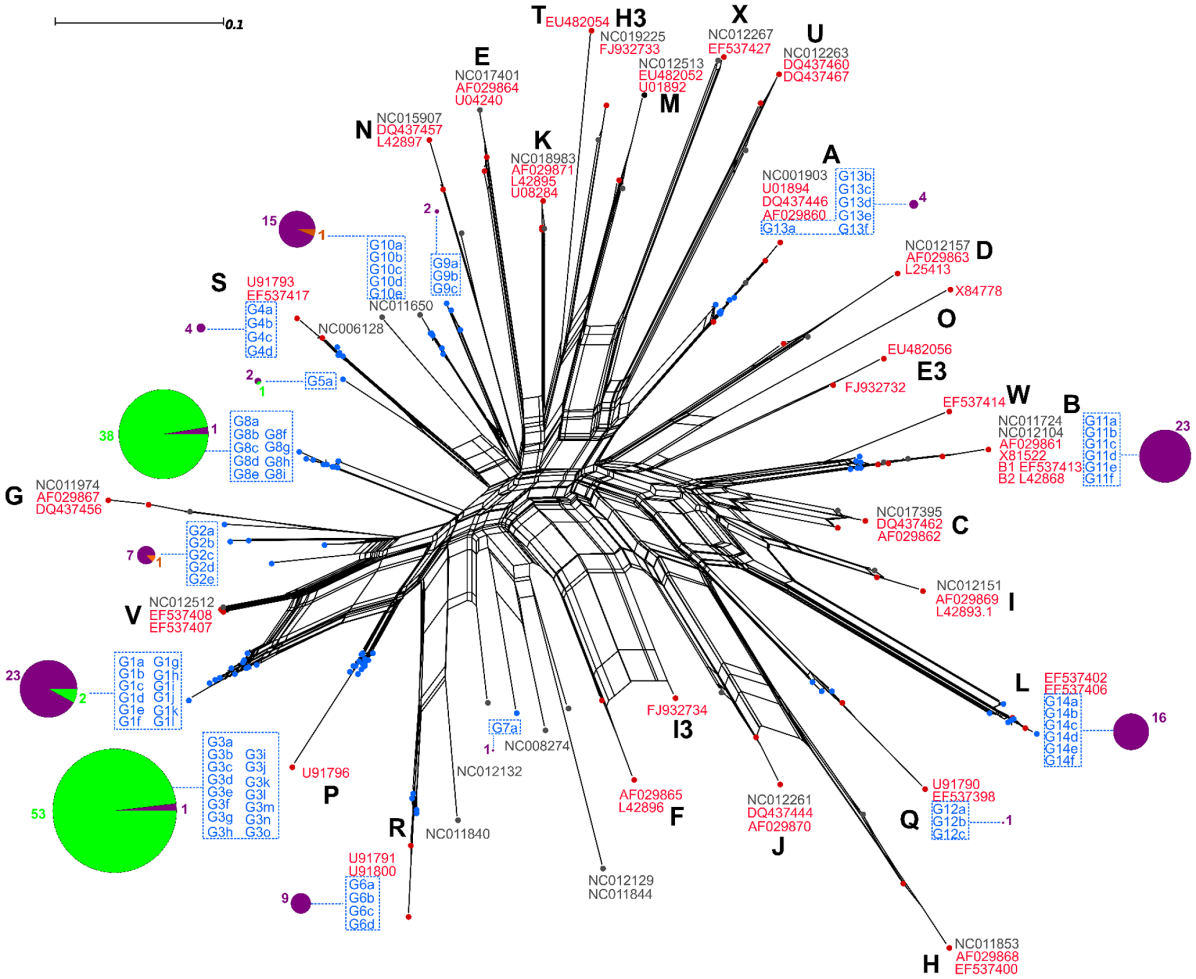
Phylogenetic network of *ospC* sequences. The network includes consensus sequences of the genotypes recovered in this study (blue) as well as reference sequences for species in the *Borrelia burgdorferi* species complex. Genotype groups are delineated by blue dotted lines, and capital letters to the side of each group represent unique *ospC* alleles detected in previous studies [37]. The pie charts connected to each main genotype group show the proportion of genotypes in the group that were isolated from each host species: Siberian chipmunks (purple), bank voles (green), or wood mice (orange).

In the *ospC* analysis, 80 genotypes were retained after grouping via the the nearest-neighbor classification algorithm. The genetic diversity displayed in the phylogenetic network of *ospC* sequences, when compared with that of the *rplB* network, was consistent with the higher number of genotypes (Figure 2). Based on this network, *ospC* genotypes were delineated into 14 groups, in which, as in the *rplB* data, there was a clear influence of host specificity. Groups G1, G2, G4, G9, G10, G11, G12, G13, and G14 included mainly sequences from chipmunks, while groups G3 and G8 consisted mainly of sequences recovered from bank voles. Sequences isolated from wood mice clustered in genotypes G2 and G10.

We studied the genetic differentiation among *Borrelia* genotypes detected in our sample using an AMOVA, in which the explanatory factors were host species, sampling year, and host individual. As expected from the phylogenetic networks, a substantial percentage of the molecular variance in our sample was explained by the different host species for both loci, 58% for *rplB* and 21% for *ospC* (Tables 1 and 2). Sampling year failed to explain much molecular variance (<1%). Finally, 11 and 17% of the observed variance could be explained by intra-individual variation in *rplB* and *ospC*, respectively.

**Table 1:** Results of the AMOVA performed on *rplB* sequence data.

Levels of variability	Df^a^	SSq^b^	MSq^c^	S^d^	%^e^	Phi^f^
Among host species	2	1039.14	519.57	0.27	58.19	0.58
Among years within host species	12	77.05	6.42	0.003	0.58	0.01
Among samples within years	172	922.79	5.37	0.14	29.83	0.72
Within samples	7072	373.20	0.053	0.053	11.41	0.89

a degrees of freedom; ^b^sums of squares; ^c^mean squares; ^d^components of covariance; ^e^percentage contribution to the total covariance; ^f^Phi statistic.

**Table 2:** Results of the AMOVA performed on *ospC* sequence data.

Levels of variability	Df^a^	SSq^b^	MSq^c^	S^d^	%^e^	Phi^f^
Among host species	2	439.44	219.72	0.11	20.93	0.21
Among years within host species	11	190.96	17.36	0.001	0.23	0.003
Among samples within years	163	2451.03	15.04	0.32	61.94	0.79
Within samples	8238	713.59	0.087	0.087	16.90	0.83

^a^degrees of freedom; ^b^sums of squares; ^c^mean squares; ^d^components of covariance; ^e^percentage contribution to the total covariance; ^f^Phi statistic.

**Figure 3:**
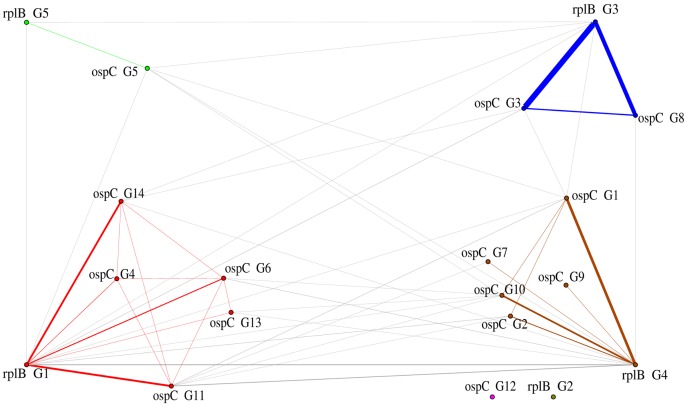
Visual representation of patterns of genotype group associations found in this study. The graph includes genotypes of both target loci and from all three host species. Each distinct community of co-occurring genotype groups is highlighted in a different color, and the thickness of lines is proportional to the frequency of co-occurrence. Vertices were placed empirically; genotypes located towards the top of the figure correspond to those mainly found in bank voles, while those towards the bottom were associated with chipmunks. The cluster of genotypes at the bottom left corresponds to strains of *B. burgdorferi* s.s. and that on the top and bottom right to *B. afzelii*.

The graph of genotype associations illustrates the frequency of co-occurrence of the different genotype groups for both markers in host individuals (Figure 3). By using measures of modularity to classify groups of co-occurring sequences, we found that the genotype groups isolated from bank voles were distinct from those isolated from chipmunks. We also found evidence that supports the delineation of various species in the *Borrelia burgdorferi* species complex. Among the various associations detected by this analysis, we observed that several *ospC* genotype groups were associated with a single *rplB* genotype. In addition, chipmunks were infected by several *rplB* genotype groups, indicating co-infection by different species of *Borrelia*. Of the individuals from which sequences were recovered, 26% of chipmunks and 11% of bank voles were co-infected by different *rplB* genotypes, while more than 30% of chipmunks, bank voles, and wood mice were co-infected by multiple *ospC* genotypes (Table 3).

**Table 3:** Percentage (%) of host individuals of each host species in which co-infection by at least two genotypes of the *Borrelia burgdorferi* species complex was detected.

Host species	*rplB*	*ospC*
*Tamias sibiricus barberi*	19.54	38.64
*Myodes glareolus*	4.55	31.33
*Apodemus sylvaticus*	0	0

Results are shown for both the *rplB* and *ospC* datasets.

## Discussion

In previous studies, genotypes of the *B. burgdorferi* species complex have been characterized using multiple techniques, including MLST using Sanger sequencing [29] [30] [31] [11] [32] and PCR-RFLP [33]. These two methods are complementary as the first gives an accurate estimation of the relatedness between bacterial genotypes and the second is more useful in detecting co-infection. Here, we used HiMLST [7] to efficiently characterize the genetic diversity of the *B. burgdorferi* species complex within three sympatric mammal species. As expected, high-throughput sequencing is potentially quite valuable in these types of studies because it combines the advantages of both MLST and PCR-RFLP.

### Benefits associated with high-throughput sequence typing

High-throughput sequencing of the *rplB* and the *ospC* markers of the *B. burgdorferi* species complex revealed a high degree of bacterial diversity within infected chipmunks, bank voles, and wood mice in the forest of Sénart (France). The observed *rplB* genotypes were analyzed in the context of pre-existing MLST data and were found to correspond to four species of *Borrelia*: i) *B. afzelii*, including a genotype group that was not described in the dedicated MLST database, ii) *B. burgdorferi* s.s., iii) *B. garinii*, and iv) *B. spielmanii*. When we used this method to assign a species label to the main genotype of each infected individual, we found that the result was very similar, but not identical, to results obtained by PCR-RFLP (Table S1, [8] [9]). An advantage of a HiMLST approach over PCR-RFLP, however, is that a more detailed description of genotypes is possible due to the increased resolution of the sequence data and the high number of sequences obtained from each sample. For example, at the *rplB* locus, we were able to identify subspecific groups within *B. afzelii*, and an even higher degree of genetic diversity was observed at the *ospC* locus. Additionally, the phylogenetic analysis based on this marker revealed that the *ospC* genotypes associated with a given host species do not form monophyletic groups, an observation that reflects the evolutionary processes, such as diversifying host-driven selective pressures and recombination, that affect this gene [6].

### Patterns of association among ospC and rplB genotypes

In spite of the fact that the observed distribution of sequence polymorphism in the *rplB* data was quite different from that of *ospC*, we were able to identify common co-occurrence patterns among the genotype groups. Some of these co-occurrence patterns described multilocus associations, i.e. multilocus sequence types, which were common within the samples studied. In particular, these patterns revealed that multiple *ospC* alleles were associated with a single allele of *rplB*, probably because of recurrent recombination in the *ospC* region. Furthermore, co-occurrence of distinct genotypes of a single locus, as we observed in the *rplB* housekeeping gene, can provide information about the co-infection of host individuals by different bacterial lineages. Statistical developments that allow researchers to quantify the relative impact of both kinds of co-occurrence patterns will be invaluable in future analyses of similar metagenomic data.

### Differences in co-infection patterns among hosts

As described above, the co-infection of individual hosts in our samples by different genotypes was not rare. For example, our graph analysis revealed the frequent presence in bank voles of two *ospC* variants genetically linked with a single *rplB* allele. In chipmunks, however, we observed more co-infection events, which can involve multiple combinations of different *Borrelia* species, a finding that could be explained by several hypotheses. First, at these study sites, chipmunks have a higher tick burden than bank voles do [34]. This could result in a higher probability of contact with bacteria of the *B. burgdorferi* species complex and, consequently, a higher risk of co-infection. Second, the chipmunk is considered an invasive species in the Sénart forest; it was introduced there in the early 1970s [35], and the species had colonized the study site by the late 1990s. An increase in the allocation of an individual's resources to reproduction at the cost of allocation to immune responses has been presented as a potential strategy of successful invasive species [36]. It might thus be that the chipmunk population established in Sénart has a low investment in immune function, resulting in a higher degree of susceptibility to infection by bacteria of the *B. burgdorferi* species complex. Finally, it is possible that more bacterial genotypes are able to infect chipmunks than bank voles.

### Differentiation among hosts

The strong genetic differentiation observed between bacterial genotypes that infected bank voles and those that infected chipmunks is compatible with the hypothesis of host-specificity in bacterial populations. In spite of the difference in polymorphism patterns between *rplB* and *ospC*, genotypes that were isolated from chipmunks and bank voles were significantly different from each other at both markers. Indeed, this study did not identify any bacterial genotype that was present at high frequencies in both host species. Our data support previous results, obtained via PCR-RFLP, that show that in a sample of chipmunks and bank voles, genotypes of *B. burgdorferi* s.s. were found only in chipmunks. However, the use of PCR-RFLP did not allow that previous study to discriminate between genotypes of *B. afzelii* associated with either chipmunks or bank voles [8]. Together with those of the current study, these results demonstrate that chipmunks and bank voles are hosts of independent genotype groups within the *B. burgdorferi* species complex. The ability of different strains of Lyme borreliosis-causing bacteria to persist in two independent host species could be explained by two non-exclusive hypotheses. First, a given host species might be able to clear infections caused by a specific subset of *Borrelia* genotypes or bacterial lineages could differ in their ability to infect the two host species, which would result in different infection cycles for the different genotypes. However, it cannot be excluded that chipmunks and bank voles are simply exposed to different bacterial genotypes as a result of host-vector interactions, a situation that could result in the creation of different, host-specific transmission cycles. For example, if ticks have a higher probability of parasitizing a given host species, some degree of differentiation in both housekeeping and virulence-related genes would likely result. It would therefore be interesting to compare samples from questing ticks and feeding ticks to determine if there are changes in the diversity of genotypes of the *B. burgdorferi* species complex.

### Origin of low frequency infections

Unlike chipmunks and bank voles, wood mice are only infrequently infected by strains of the *B. burgdorferi* species complex [8], and all but one of the genotypes identified from wood mice were shared with the two other host species. This finding could indicate that wood mice are able to become infected but do not play a critical role in the dynamic of the *B. burgdorferi* species complex in Sénart forest. This infection pattern in wood mice might be due to either i) a relative paucity of bacterial strains that are adapted to infect wood mice, or ii) the low susceptibility to *Borrelia* of wood mice in Sénart. The infection of a few chipmunks and bank voles by atypical (e.g., *B. garinii*) and/or low frequency genotypes might illustrate similar infection patterns.

### Perspectives

To conclude, this study used high-throughput sequencing to reveal that strains of the *Borrelia burgdorferi* species complex that were isolated from three sympatric rodent host species presented strong evidence of genetic differentiation at two different loci subject to distinct evolutionary pressures. This result strengthens the need of experimental studies to investigate properly the adaptation of host specific lineages to assess more accurately the dynamic of Lyme disease bacteria among hosts. The efficiency of the HiMLST procedure opens new avenues of study and offers exciting opportunities for further characterization of the diversity and evolution of pathogens infecting host species and vectors. It is indeed important to take into account the variability of pathogens within samples to i) assess properly their population genetic structure and, ii) compare the diversity among infected individuals and eventually at a tissue scale within individuals.

## Supporting Information

Table S1 Information on the host individuals sampled and the identity of the *Borrelia* species that were detected in each sample via PCR-RFLP [8] [9] and/or HiMLST. Species labels were assigned to genotypes in this study based on the genotype groups present in the *rplB* data. Genotype G1 was assigned to *Borrelia burgdorferi* s.s., G3 and G4 to *Borrelia afzelii*, and G5 to *Borrelia garinii*. ^0^means that the species was not detected by PCR-RFLP or HiMLST; ^1^that the species was detected only by PCR-RFLP; ^2^that species was detected only by HiMLST; and ^3^species was detected by both methods; ^X^no sequences were retained for this individual after genotypes containing fewer than four sequences were removed.
(DOC)
