# Supplementary material for: High-Throughput Sequence Typing Reveals Genetic Differentiation and Host Specialization among Populations of the Borrelia burgdorferi Species Complex that Infect Rodents
Source: PLoS One. 2014 Feb 12;9(2):e88581. doi: 10.1371/journal.pone.0088581 (PMC3922933; doi:10.1371/journal.pone.0088581)
Supplement: Table S1 — Information on the host individuals sampled and the identity of the Borrelia species that were detected in each sample via PCR-RFLP [8] [9] and/or HiMLST. Species labels were assigned to genotypes in this study based on the genotype groups present in the rplB data. Genotype G1 was assigned to Borrelia burgdorferi s.s., G3 and G4 to Borrelia afzelii, and G5 to Borrelia garinii. 0means that the species was not detected by PCR-RFLP or HiMLST; 1that the species was detected only by PCR-RFLP; 2that species was detected only by HiMLST; and 3species was detected by both methods; Xno sequences were retained for this individual after genotypes containing fewer than four sequences were removed. (DOC) [file pone.0088581.s001.doc]

| - - 1. Individual name | - - 1. Host species | - - 1. Sampling year | - - 1. *B. afzelii* | - - 1. *B. burgdorferi s. s.* | - - 1. *B. garinii* |
| --- | --- | --- | --- | --- | --- |
| - - 1. S05Cg006 | - - 1. *Myodes glareolus* | - - 1. 2005 | - - 1. 3 | - - 1. 0 | - - 1. 0 |
| - - 1. S05Cg008 | - - 1. *Myodes glareolus* | - - 1. 2005 | - - 1. 3 | - - 1. 0 | - - 1. 0 |
| - - 1. S05Cg029a | - - 1. *Myodes glareolus* | - - 1. 2005 | - - 1. 3 | - - 1. 0 | - - 1. 0 |
| - - 1. S05Cg032 | - - 1. *Myodes glareolus* | - - 1. 2005 | - - 1. 3 | - - 1. 0 | - - 1. 0 |
| - - 1. S05Cg048 | - - 1. *Myodes glareolus* | - - 1. 2005 | - - 1. 3 | - - 1. 0 | - - 1. 0 |
| - - 1. S05Cg051 | - - 1. *Myodes glareolus* | - - 1. 2005 | - - 1. 1 | - - 1. 2 | - - 1. 0 |
| - - 1. S05Cg057 | - - 1. *Myodes glareolus* | - - 1. 2005 | - - 1. 3 | - - 1. 0 | - - 1. 0 |
| - - 1. S05Cg060 | - - 1. *Myodes glareolus* | - - 1. 2005 | - - 1. X | - - 1. X | - - 1. X |
| - - 1. S05Cg062 | - - 1. *Myodes glareolus* | - - 1. 2005 | - - 1. X | - - 1. X | - - 1. X |
| - - 1. S06Cg001 | - - 1. *Myodes glareolus* | - - 1. 2006 | - - 1. 3 | - - 1. 0 | - - 1. 0 |
| - - 1. S06Cg012 | - - 1. *Myodes glareolus* | - - 1. 2006 | - - 1. 3 | - - 1. 0 | - - 1. 0 |
| - - 1. S06Cg017 | - - 1. *Myodes glareolus* | - - 1. 2006 | - - 1. 3 | - - 1. 0 | - - 1. 0 |
| - - 1. S06Cg022 | - - 1. *Myodes glareolus* | - - 1. 2006 | - - 1. 3 | - - 1. 0 | - - 1. 0 |
| - - 1. S06Cg023 | - - 1. *Myodes glareolus* | - - 1. 2006 | - - 1. 3 | - - 1. 0 | - - 1. 0 |
| - - 1. S06Cg025 | - - 1. *Myodes glareolus* | - - 1. 2006 | - - 1. 3 | - - 1. 0 | - - 1. 0 |
| - - 1. S06Cg038 | - - 1. *Myodes glareolus* | - - 1. 2006 | - - 1. 3 | - - 1. 0 | - - 1. 0 |
| - - 1. S06Cg041 | - - 1. *Myodes glareolus* | - - 1. 2006 | - - 1. 3 | - - 1. 0 | - - 1. 0 |
| - - 1. S06Cg043 | - - 1. *Myodes glareolus* | - - 1. 2006 | - - 1. 3 | - - 1. 0 | - - 1. 0 |
| - - 1. S06Cg044 | - - 1. *Myodes glareolus* | - - 1. 2006 | - - 1. 3 | - - 1. 0 | - - 1. 0 |
| - - 1. S06Cg045 | - - 1. *Myodes glareolus* | - - 1. 2006 | - - 1. 3 | - - 1. 0 | - - 1. 0 |
| - - 1. S06Cg048 | - - 1. *Myodes glareolus* | - - 1. 2006 | - - 1. 3 | - - 1. 0 | - - 1. 0 |
| - - 1. S06Cg051 | - - 1. *Myodes glareolus* | - - 1. 2006 | - - 1. 3 | - - 1. 0 | - - 1. 0 |
| - - 1. S06Cg059 | - - 1. *Myodes glareolus* | - - 1. 2006 | - - 1. 3 | - - 1. 0 | - - 1. 0 |
| - - 1. S06Cg060 | - - 1. *Myodes glareolus* | - - 1. 2006 | - - 1. 3 | - - 1. 0 | - - 1. 0 |
| - - 1. S06Cg061 | - - 1. *Myodes glareolus* | - - 1. 2006 | - - 1. 3 | - - 1. 0 | - - 1. 0 |
| - - 1. S06Cg074 | - - 1. *Myodes glareolus* | - - 1. 2006 | - - 1. 3 | - - 1. 0 | - - 1. 0 |
| - - 1. S07Cg003 | - - 1. *Myodes glareolus* | - - 1. 2007 | - - 1. 3 | - - 1. 0 | - - 1. 0 |
| - - 1. S07Cg013 | - - 1. *Myodes glareolus* | - - 1. 2007 | - - 1. 3 | - - 1. 0 | - - 1. 0 |
| - - 1. S07Cg045 | - - 1. *Myodes glareolus* | - - 1. 2007 | - - 1. 3 | - - 1. 0 | - - 1. 0 |
| - - 1. S07Cg055 | - - 1. *Myodes glareolus* | - - 1. 2007 | - - 1. 3 | - - 1. 0 | - - 1. 0 |
| - - 1. S07Cg090 | - - 1. *Myodes glareolus* | - - 1. 2007 | - - 1. 3 | - - 1. 0 | - - 1. 0 |
| - - 1. S07Cg093 | - - 1. *Myodes glareolus* | - - 1. 2007 | - - 1. 3 | - - 1. 0 | - - 1. 0 |
| - - 1. S07Cg094 | - - 1. *Myodes glareolus* | - - 1. 2007 | - - 1. 3 | - - 1. 0 | - - 1. 0 |
| - - 1. S07Cg97 | - - 1. *Myodes glareolus* | - - 1. 2007 | - - 1. X | - - 1. X | - - 1. X |
| - - 1. S07Cg098 | - - 1. *Myodes glareolus* | - - 1. 2007 | - - 1. 3 | - - 1. 0 | - - 1. 0 |
| - - 1. S07Cg101 | - - 1. *Myodes glareolus* | - - 1. 2007 | - - 1. 3 | - - 1. 0 | - - 1. 0 |
| - - 1. S07Cg105 | - - 1. *Myodes glareolus* | - - 1. 2007 | - - 1. 3 | - - 1. 0 | - - 1. 0 |
| - - 1. S07Cg112 | - - 1. *Myodes glareolus* | - - 1. 2007 | - - 1. 3 | - - 1. 0 | - - 1. 0 |
| - - 1. S07Cg113 | - - 1. *Myodes glareolus* | - - 1. 2007 | - - 1. 3 | - - 1. 0 | - - 1. 0 |
| - - 1. S07Cg117 | - - 1. *Myodes glareolus* | - - 1. 2007 | - - 1. 3 | - - 1. 0 | - - 1. 0 |
| - - 1. S07Cg123 | - - 1. *Myodes glareolus* | - - 1. 2007 | - - 1. 3 | - - 1. 0 | - - 1. 0 |
| - - 1. S07Cg132 | - - 1. *Myodes glareolus* | - - 1. 2007 | - - 1. 3 | - - 1. 0 | - - 1. 0 |
| - - 1. S07Cg149 | - - 1. *Myodes glareolus* | - - 1. 2007 | - - 1. 3 | - - 1. 0 | - - 1. 0 |
| - - 1. S07Cg156 | - - 1. *Myodes glareolus* | - - 1. 2007 | - - 1. 3 | - - 1. 0 | - - 1. 0 |
| - - 1. S07Cg158 | - - 1. *Myodes glareolus* | - - 1. 2007 | - - 1. 3 | - - 1. 0 | - - 1. 0 |
| - - 1. S07Cg164 | - - 1. *Myodes glareolus* | - - 1. 2007 | - - 1. 3 | - - 1. 0 | - - 1. 0 |
| - - 1. S07Cg175 | - - 1. *Myodes glareolus* | - - 1. 2007 | - - 1. 3 | - - 1. 0 | - - 1. 0 |
| - - 1. S07Cg219 | - - 1. *Myodes glareolus* | - - 1. 2007 | - - 1. X | - - 1. X | - - 1. X |
| - - 1. S07Cg221 | - - 1. *Myodes glareolus* | - - 1. 2007 | - - 1. 3 | - - 1. 0 | - - 1. 0 |
| - - 1. S07Cg226 | - - 1. *Myodes glareolus* | - - 1. 2007 | - - 1. 3 | - - 1. 0 | - - 1. 0 |
| - - 1. S07Cg231 | - - 1. *Myodes glareolus* | - - 1. 2007 | - - 1. 3 | - - 1. 0 | - - 1. 0 |
| - - 1. S07Cg247 | - - 1. *Myodes glareolus* | - - 1. 2007 | - - 1. 3 | - - 1. 0 | - - 1. 0 |
| - - 1. S07Cg262 | - - 1. *Myodes glareolus* | - - 1. 2007 | - - 1. 3 | - - 1. 0 | - - 1. 0 |
| - - 1. S07Cg267 | - - 1. *Myodes glareolus* | - - 1. 2007 | - - 1. 3 | - - 1. 0 | - - 1. 0 |
| - - 1. S07Cg279 | - - 1. *Myodes glareolus* | - - 1. 2007 | - - 1. 3 | - - 1. 0 | - - 1. 0 |
| - - 1. S07Cg286 | - - 1. *Myodes glareolus* | - - 1. 2007 | - - 1. 3 | - - 1. 0 | - - 1. 0 |
| - - 1. S07Cg292 | - - 1. *Myodes glareolus* | - - 1. 2007 | - - 1. 3 | - - 1. 0 | - - 1. 0 |
| - - 1. S07Cg295 | - - 1. *Myodes glareolus* | - - 1. 2007 | - - 1. 3 | - - 1. 0 | - - 1. 0 |
| - - 1. S07Cg299 | - - 1. *Myodes glareolus* | - - 1. 2007 | - - 1. 3 | - - 1. 0 | - - 1. 0 |
| - - 1. S07Cg302 | - - 1. *Myodes glareolus* | - - 1. 2007 | - - 1. 3 | - - 1. 0 | - - 1. 0 |
| - - 1. S07Cg311 | - - 1. *Myodes glareolus* | - - 1. 2007 | - - 1. 3 | - - 1. 0 | - - 1. 0 |
| - - 1. S07Cg315 | - - 1. *Myodes glareolus* | - - 1. 2007 | - - 1. 3 | - - 1. 0 | - - 1. 0 |
| - - 1. S07Cg324 | - - 1. *Myodes glareolus* | - - 1. 2007 | - - 1. 3 | - - 1. 0 | - - 1. 0 |
| - - 1. S07Cg330 | - - 1. *Myodes glareolus* | - - 1. 2007 | - - 1. 3 | - - 1. 0 | - - 1. 0 |
| - - 1. S07Cg334 | - - 1. *Myodes glareolus* | - - 1. 2007 | - - 1. 3 | - - 1. 0 | - - 1. 0 |
| - - 1. S07Cg337 | - - 1. *Myodes glareolus* | - - 1. 2007 | - - 1. 3 | - - 1. 0 | - - 1. 0 |
| - - 1. S08Cg101 | - - 1. *Myodes glareolus* | - - 1. 2008 | - - 1. 3 | - - 1. 0 | - - 1. 0 |
| - - 1. S08Cg102 | - - 1. *Myodes glareolus* | - - 1. 2008 | - - 1. 3 | - - 1. 0 | - - 1. 0 |
| - - 1. S08Cg103 | - - 1. *Myodes glareolus* | - - 1. 2008 | - - 1. 3 | - - 1. 0 | - - 1. 0 |
| - - 1. S08Cg104 | - - 1. *Myodes glareolus* | - - 1. 2008 | - - 1. 3 | - - 1. 0 | - - 1. 0 |
| - - 1. S08Cg109 | - - 1. *Myodes glareolus* | - - 1. 2008 | - - 1. 3 | - - 1. 0 | - - 1. 0 |
| - - 1. S08Cg111 | - - 1. *Myodes glareolus* | - - 1. 2008 | - - 1. 3 | - - 1. 0 | - - 1. 0 |
| - - 1. S08Cg113 | - - 1. *Myodes glareolus* | - - 1. 2008 | - - 1. 3 | - - 1. 0 | - - 1. 0 |
| - - 1. S08Cg115 | - - 1. *Myodes glareolus* | - - 1. 2008 | - - 1. 3 | - - 1. 0 | - - 1. 0 |
| - - 1. S08Cg121 | - - 1. *Myodes glareolus* | - - 1. 2008 | - - 1. 3 | - - 1. 0 | - - 1. 0 |
| - - 1. S08Cg132 | - - 1. *Myodes glareolus* | - - 1. 2008 | - - 1. 3 | - - 1. 0 | - - 1. 0 |
| - - 1. S08Cg178 | - - 1. *Myodes glareolus* | - - 1. 2008 | - - 1. 3 | - - 1. 0 | - - 1. 0 |
| - - 1. S08Cg181 | - - 1. *Myodes glareolus* | - - 1. 2008 | - - 1. X | - - 1. X | - - 1. X |
| - - 1. S08Cg196 | - - 1. *Myodes glareolus* | - - 1. 2008 | - - 1. 3 | - - 1. 0 | - - 1. 0 |
| - - 1. S08Cg221 | - - 1. *Myodes glareolus* | - - 1. 2008 | - - 1. 3 | - - 1. 0 | - - 1. 0 |
| - - 1. S08Cg226 | - - 1. *Myodes glareolus* | - - 1. 2008 | - - 1. 3 | - - 1. 0 | - - 1. 0 |
| - - 1. S08Cg235 | - - 1. *Myodes glareolus* | - - 1. 2008 | - - 1. 3 | - - 1. 0 | - - 1. 0 |
| - - 1. S08Cg251 | - - 1. *Myodes glareolus* | - - 1. 2008 | - - 1. 3 | - - 1. 0 | - - 1. 0 |
| - - 1. S08Cg349 | - - 1. *Myodes glareolus* | - - 1. 2008 | - - 1. 3 | - - 1. 0 | - - 1. 0 |
| - - 1. S08Cg383 | - - 1. *Myodes glareolus* | - - 1. 2008 | - - 1. 3 | - - 1. 0 | - - 1. 0 |
| - - 1. S08Cg384 | - - 1. *Myodes glareolus* | - - 1. 2008 | - - 1. 3 | - - 1. 0 | - - 1. 0 |
| - - 1. S08Cg391 | - - 1. *Myodes glareolus* | - - 1. 2008 | - - 1. 3 | - - 1. 0 | - - 1. 0 |
| - - 1. S08Cg395 | - - 1. *Myodes glareolus* | - - 1. 2008 | - - 1. 3 | - - 1. 0 | - - 1. 0 |
| - - 1. S08Cg396 | - - 1. *Myodes glareolus* | - - 1. 2008 | - - 1. 3 | - - 1. 0 | - - 1. 0 |
| - - 1. S09Cg004 | - - 1. *Myodes glareolus* | - - 1. 2009 | - - 1. 3 | - - 1. 0 | - - 1. 3 |
| - - 1. S09Cg006 | - - 1. *Myodes glareolus* | - - 1. 2009 | - - 1. 3 | - - 1. 0 | - - 1. 0 |
| - - 1. S10Cg023 | - - 1. *Myodes glareolus* | - - 1. 2010 | - - 1. 3 | - - 1. 0 | - - 1. 0 |
| - - 1. S10Cg064 | - - 1. *Myodes glareolus* | - - 1. 2010 | - - 1. X | - - 1. X | - - 1. X |
| - - 1. S05Ts002 | - - 1. *Tamias sibiricus* | - - 1. 2005 | - - 1. 3 | - - 1. 0 | - - 1. 0 |
| - - 1. S05Ts014 | - - 1. *Tamias sibiricus* | - - 1. 2005 | - - 1. 3 | - - 1. 0 | - - 1. 0 |
| - - 1. S05Ts017 | - - 1. *Tamias sibiricus* | - - 1. 2005 | - - 1. 3 | - - 1. 0 | - - 1. 0 |
| - - 1. S05Ts018 | - - 1. *Tamias sibiricus* | - - 1. 2005 | - - 1. 1 | - - 1. 3 | - - 1. 0 |
| - - 1. S05Ts023 | - - 1. *Tamias sibiricus* | - - 1. 2005 | - - 1. 0 | - - 1. 3 | - - 1. 0 |
| - - 1. S05Ts024 | - - 1. *Tamias sibiricus* | - - 1. 2005 | - - 1. 3 | - - 1. 2 | - - 1. 0 |
| - - 1. S05Ts032 | - - 1. *Tamias sibiricus* | - - 1. 2005 | - - 1. 3 | - - 1. 0 | - - 1. 0 |
| - - 1. S05Ts034 | - - 1. *Tamias sibiricus* | - - 1. 2005 | - - 1. 3 | - - 1. 0 | - - 1. 0 |
| - - 1. S06Ts001 | - - 1. *Tamias sibiricus* | - - 1. 2006 | - - 1. X | - - 1. X | - - 1. X |
| - - 1. S06Ts003 | - - 1. *Tamias sibiricus* | - - 1. 2006 | - - 1. 3 | - - 1. 2 | - - 1. 0 |
| - - 1. S06Ts008 | - - 1. *Tamias sibiricus* | - - 1. 2006 | - - 1. X | - - 1. X | - - 1. X |
| - - 1. S06Ts010 | - - 1. *Tamias sibiricus* | - - 1. 2006 | - - 1. X | - - 1. X | - - 1. X |
| - - 1. S06Ts014 | - - 1. *Tamias sibiricus* | - - 1. 2006 | - - 1. 0 | - - 1. 3 | - - 1. 0 |
| - - 1. S06Ts015 | - - 1. *Tamias sibiricus* | - - 1. 2006 | - - 1. 0 | - - 1. 3 | - - 1. 0 |
| - - 1. S06Ts016 | - - 1. *Tamias sibiricus* | - - 1. 2006 | - - 1. X | - - 1. X | - - 1. X |
| - - 1. S06Ts017 | - - 1. *Tamias sibiricus* | - - 1. 2006 | - - 1. 0 | - - 1. 3 | - - 1. 0 |
| - - 1. S06Ts018 | - - 1. *Tamias sibiricus* | - - 1. 2006 | - - 1. 3 | - - 1. 0 | - - 1. 0 |
| - - 1. S06Ts026 | - - 1. *Tamias sibiricus* | - - 1. 2006 | - - 1. X | - - 1. X | - - 1. X |
| - - 1. S06Ts035 | - - 1. *Tamias sibiricus* | - - 1. 2006 | - - 1. 3 | - - 1. 0 | - - 1. 0 |
| - - 1. S06Ts061 | - - 1. *Tamias sibiricus* | - - 1. 2006 | - - 1. X | - - 1. X | - - 1. X |
| - - 1. S06Ts063 | - - 1. *Tamias sibiricus* | - - 1. 2006 | - - 1. X | - - 1. X | - - 1. X |
| - - 1. S07Ts003 | - - 1. *Tamias sibiricus* | - - 1. 2007 | - - 1. 0 | - - 1. 3 | - - 1. 0 |
| - - 1. S07Ts004 | - - 1. *Tamias sibiricus* | - - 1. 2007 | - - 1. 0 | - - 1. 3 | - - 1. 0 |
| - - 1. S07Ts005 | - - 1. *Tamias sibiricus* | - - 1. 2007 | - - 1. X | - - 1. X | - - 1. X |
| - - 1. S07Ts006 | - - 1. *Tamias sibiricus* | - - 1. 2007 | - - 1. 3 | - - 1. 0 | - - 1. 0 |
| - - 1. S07Ts009 | - - 1. *Tamias sibiricus* | - - 1. 2007 | - - 1. 3 | - - 1. 0 | - - 1. 0 |
| - - 1. S07Ts012 | - - 1. *Tamias sibiricus* | - - 1. 2007 | - - 1. X | - - 1. X | - - 1. X |
| - - 1. S07Ts015 | - - 1. *Tamias sibiricus* | - - 1. 2007 | - - 1. X | - - 1. X | - - 1. X |
| - - 1. S07Ts019 | - - 1. *Tamias sibiricus* | - - 1. 2007 | - - 1. 0 | - - 1. 3 | - - 1. 0 |
| - - 1. S07Ts023 | - - 1. *Tamias sibiricus* | - - 1. 2007 | - - 1. 3 | - - 1. 0 | - - 1. 0 |
| - - 1. S07Ts025 | - - 1. *Tamias sibiricus* | - - 1. 2007 | - - 1. 3 | - - 1. 0 | - - 1. 0 |
| - - 1. S07Ts027 | - - 1. *Tamias sibiricus* | - - 1. 2007 | - - 1. 0 | - - 1. 3 | - - 1. 0 |
| - - 1. S07Ts031 | - - 1. *Tamias sibiricus* | - - 1. 2007 | - - 1. 3 | - - 1. 0 | - - 1. 0 |
| - - 1. S07Ts043 | - - 1. *Tamias sibiricus* | - - 1. 2007 | - - 1. 2 | - - 1. 3 | - - 1. 0 |
| - - 1. S07Ts051 | - - 1. *Tamias sibiricus* | - - 1. 2007 | - - 1. X | - - 1. X | - - 1. X |
| - - 1. S07Ts055 | - - 1. *Tamias sibiricus* | - - 1. 2007 | - - 1. 0 | - - 1. 3 | - - 1. 0 |
| - - 1. S07Ts056 | - - 1. *Tamias sibiricus* | - - 1. 2007 | - - 1. 0 | - - 1. 3 | - - 1. 0 |
| - - 1. S07Ts057 | - - 1. *Tamias sibiricus* | - - 1. 2007 | - - 1. 0 | - - 1. 3 | - - 1. 0 |
| - - 1. S07Ts059 | - - 1. *Tamias sibiricus* | - - 1. 2007 | - - 1. 0 | - - 1. 3 | - - 1. 0 |
| - - 1. S07Ts061 | - - 1. *Tamias sibiricus* | - - 1. 2007 | - - 1. X | - - 1. X | - - 1. X |
| - - 1. S07Ts064 | - - 1. *Tamias sibiricus* | - - 1. 2007 | - - 1. 2 | - - 1. 3 | - - 1. 0 |
| - - 1. S07Ts066 | - - 1. *Tamias sibiricus* | - - 1. 2007 | - - 1. 3 | - - 1. 0 | - - 1. 0 |
| - - 1. S07Ts068 | - - 1. *Tamias sibiricus* | - - 1. 2007 | - - 1. 3 | - - 1. 0 | - - 1. 0 |
| - - 1. S07Ts069 | - - 1. *Tamias sibiricus* | - - 1. 2007 | - - 1. 3 | - - 1. 0 | - - 1. 0 |
| - - 1. S07Ts070 | - - 1. *Tamias sibiricus* | - - 1. 2007 | - - 1. 3 | - - 1. 0 | - - 1. 0 |
| - - 1. S07Ts073 | - - 1. *Tamias sibiricus* | - - 1. 2007 | - - 1. 3 | - - 1. 2 | - - 1. 1 |
| - - 1. S07Ts076 | - - 1. *Tamias sibiricus* | - - 1. 2007 | - - 1. 0 | - - 1. 3 | - - 1. 0 |
| - - 1. S07Ts078 | - - 1. *Tamias sibiricus* | - - 1. 2007 | - - 1. 3 | - - 1. 0 | - - 1. 0 |
| - - 1. S07Ts079 | - - 1. *Tamias sibiricus* | - - 1. 2007 | - - 1. 3 | - - 1. 0 | - - 1. 0 |
| - - 1. S07Ts081 | - - 1. *Tamias sibiricus* | - - 1. 2007 | - - 1. 3 | - - 1. 0 | - - 1. 0 |
| - - 1. S07Ts082 | - - 1. *Tamias sibiricus* | - - 1. 2007 | - - 1. 0 | - - 1. 2 | - - 1. 3 |
| - - 1. S07Ts084 | - - 1. *Tamias sibiricus* | - - 1. 2007 | - - 1. X | - - 1. X | - - 1. X |
| - - 1. S07Ts088 | - - 1. *Tamias sibiricus* | - - 1. 2007 | - - 1. X | - - 1. X | - - 1. X |
| - - 1. S07Ts089 | - - 1. *Tamias sibiricus* | - - 1. 2007 | - - 1. 0 | - - 1. 3 | - - 1. 0 |
| - - 1. S07Ts090 | - - 1. *Tamias sibiricus* | - - 1. 2007 | - - 1. 0 | - - 1. 3 | - - 1. 0 |
| - - 1. S07Ts091 | - - 1. *Tamias sibiricus* | - - 1. 2007 | - - 1. 3 | - - 1. 0 | - - 1. 0 |
| - - 1. S07Ts092 | - - 1. *Tamias sibiricus* | - - 1. 2007 | - - 1. 0 | - - 1. 3 | - - 1. 0 |
| - - 1. S07Ts098 | - - 1. *Tamias sibiricus* | - - 1. 2007 | - - 1. 3 | - - 1. 0 | - - 1. 0 |
| - - 1. S07Ts099 | - - 1. *Tamias sibiricus* | - - 1. 2007 | - - 1. 0 | - - 1. 3 | - - 1. 0 |
| - - 1. S07Ts100 | - - 1. *Tamias sibiricus* | - - 1. 2007 | - - 1. 0 | - - 1. 3 | - - 1. 0 |
| - - 1. S07Ts103 | - - 1. *Tamias sibiricus* | - - 1. 2007 | - - 1. X | - - 1. X | - - 1. X |
| - - 1. S07Ts107 | - - 1. *Tamias sibiricus* | - - 1. 2007 | - - 1. 0 | - - 1. 3 | - - 1. 0 |
| - - 1. S08Ts014 | - - 1. *Tamias sibiricus* | - - 1. 2008 | - - 1. 0 | - - 1. 3 | - - 1. 0 |
| - - 1. S08Ts031 | - - 1. *Tamias sibiricus* | - - 1. 2008 | - - 1. X | - - 1. X | - - 1. X |
| - - 1. S08Ts035 | - - 1. *Tamias sibiricus* | - - 1. 2008 | - - 1. 2 | - - 1. 2 | - - 1. 1 |
| - - 1. S08Ts043 | - - 1. *Tamias sibiricus* | - - 1. 2008 | - - 1. 0 | - - 1. 3 | - - 1. 0 |
| - - 1. S08Ts061 | - - 1. *Tamias sibiricus* | - - 1. 2008 | - - 1. X | - - 1. X | - - 1. X |
| - - 1. S08Ts066 | - - 1. *Tamias sibiricus* | - - 1. 2008 | - - 1. 0 | - - 1. 2 | - - 1. 1 |
| - - 1. S08Ts083 | - - 1. *Tamias sibiricus* | - - 1. 2008 | - - 1. 0 | - - 1. 3 | - - 1. 0 |
| - - 1. S08Ts088 | - - 1. *Tamias sibiricus* | - - 1. 2008 | - - 1. X | - - 1. X | - - 1. X |
| - - 1. S08Ts098 | - - 1. *Tamias sibiricus* | - - 1. 2008 | - - 1. 0 | - - 1. 3 | - - 1. 0 |
| - - 1. S08Ts100 | - - 1. *Tamias sibiricus* | - - 1. 2008 | - - 1. 0 | - - 1. 3 | - - 1. 0 |
| - - 1. S08Ts113 | - - 1. *Tamias sibiricus* | - - 1. 2008 | - - 1. 2 | - - 1. 1 | - - 1. 0 |
| - - 1. S08Ts120 | - - 1. *Tamias sibiricus* | - - 1. 2008 | - - 1. 3 | - - 1. 0 | - - 1. 0 |
| - - 1. S08Ts124 | - - 1. *Tamias sibiricus* | - - 1. 2008 | - - 1. 1 | - - 1. 0 | - - 1. 0 |
| - - 1. S08Ts125 | - - 1. *Tamias sibiricus* | - - 1. 2008 | - - 1. 0 | - - 1. 3 | - - 1. 0 |
| - - 1. S08Ts128 | - - 1. *Tamias sibiricus* | - - 1. 2008 | - - 1. 2 | - - 1. 1 | - - 1. 0 |
| - - 1. S08Ts136 | - - 1. *Tamias sibiricus* | - - 1. 2008 | - - 1. 3 | - - 1. 0 | - - 1. 0 |
| - - 1. S08Ts139 | - - 1. *Tamias sibiricus* | - - 1. 2008 | - - 1. 3 | - - 1. 0 | - - 1. 0 |
| - - 1. S08Ts146 | - - 1. *Tamias sibiricus* | - - 1. 2008 | - - 1. 2 | - - 1. 1 | - - 1. 0 |
| - - 1. S08Ts148 | - - 1. *Tamias sibiricus* | - - 1. 2008 | - - 1. 0 | - - 1. 3 | - - 1. 0 |
| - - 1. S08Ts150 | - - 1. *Tamias sibiricus* | - - 1. 2008 | - - 1. 3 | - - 1. 0 | - - 1. 0 |
| - - 1. S08Ts151 | - - 1. *Tamias sibiricus* | - - 1. 2008 | - - 1. 3 | - - 1. 0 | - - 1. 0 |
| - - 1. S08Ts156 | - - 1. *Tamias sibiricus* | - - 1. 2008 | - - 1. 0 | - - 1. 3 | - - 1. 0 |
| - - 1. S08Ts158 | - - 1. *Tamias sibiricus* | - - 1. 2008 | - - 1. 3 | - - 1. 0 | - - 1. 0 |
| - - 1. S08Ts159 | - - 1. *Tamias sibiricus* | - - 1. 2008 | - - 1. 3 | - - 1. 0 | - - 1. 0 |
| - - 1. S08Ts161 | - - 1. *Tamias sibiricus* | - - 1. 2008 | - - 1. 2 | - - 1. 1 | - - 1. 0 |
| - - 1. S08Ts166 | - - 1. *Tamias sibiricus* | - - 1. 2008 | - - 1. 3 | - - 1. 2 | - - 1. 0 |
| - - 1. S08Ts167 | - - 1. *Tamias sibiricus* | - - 1. 2008 | - - 1. 0 | - - 1. 3 | - - 1. 0 |
| - - 1. S08Ts176 | - - 1. *Tamias sibiricus* | - - 1. 2008 | - - 1. 2 | - - 1. 3 | - - 1. 0 |
| - - 1. S08Ts178 | - - 1. *Tamias sibiricus* | - - 1. 2008 | - - 1. 0 | - - 1. 3 | - - 1. 0 |
| - - 1. S08Ts186 | - - 1. *Tamias sibiricus* | - - 1. 2008 | - - 1. 0 | - - 1. 3 | - - 1. 0 |
| - - 1. S09Ts060 | - - 1. *Tamias sibiricus* | - - 1. 2009 | - - 1. X | - - 1. X | - - 1. X |
| - - 1. S09Ts108 | - - 1. *Tamias sibiricus* | - - 1. 2009 | - - 1. X | - - 1. X | - - 1. X |
| - - 1. S09Ts109 | - - 1. *Tamias sibiricus* | - - 1. 2009 | - - 1. 3 | - - 1. 1 | - - 1. 0 |
| - - 1. S09Ts155 | - - 1. *Tamias sibiricus* | - - 1. 2009 | - - 1. X | - - 1. X | - - 1. X |
| - - 1. S09Ts156 | - - 1. *Tamias sibiricus* | - - 1. 2009 | - - 1. 3 | - - 1. 0 | - - 1. 0 |
| - - 1. S09Ts158 | - - 1. *Tamias sibiricus* | - - 1. 2009 | - - 1. X | - - 1. X | - - 1. X |
| - - 1. S09Ts159 | - - 1. *Tamias sibiricus* | - - 1. 2009 | - - 1. X | - - 1. X | - - 1. X |
| - - 1. S09Ts160 | - - 1. *Tamias sibiricus* | - - 1. 2009 | - - 1. X | - - 1. X | - - 1. X |
| - - 1. S09Ts180 | - - 1. *Tamias sibiricus* | - - 1. 2009 | - - 1. X | - - 1. X | - - 1. X |
| - - 1. S09Ts180 | - - 1. *Tamias sibiricus* | - - 1. 2009 | - - 1. X | - - 1. X | - - 1. X |
| - - 1. S09Ts187 | - - 1. *Tamias sibiricus* | - - 1. 2009 | - - 1. X | - - 1. X | - - 1. X |
| - - 1. S09Ts196 | - - 1. *Tamias sibiricus* | - - 1. 2009 | - - 1. X | - - 1. X | - - 1. X |
| - - 1. S09Ts204 | - - 1. *Tamias sibiricus* | - - 1. 2009 | - - 1. 3 | - - 1. 0 | - - 1. 0 |
| - - 1. S09Ts214 | - - 1. *Tamias sibiricus* | - - 1. 2009 | - - 1. X | - - 1. X | - - 1. X |
| - - 1. S10Ts018 | - - 1. *Tamias sibiricus* | - - 1. 2010 | - - 1. X | - - 1. X | - - 1. X |
| - - 1. S10Ts029 | - - 1. *Tamias sibiricus* | - - 1. 2010 | - - 1. X | - - 1. X | - - 1. X |
| - - 1. S10Ts039 | - - 1. *Tamias sibiricus* | - - 1. 2010 | - - 1. X | - - 1. X | - - 1. X |
| - - 1. S10Ts041 | - - 1. *Tamias sibiricus* | - - 1. 2010 | - - 1. X | - - 1. X | - - 1. X |
| - - 1. S10Ts062 | - - 1. *Tamias sibiricus* | - - 1. 2010 | - - 1. X | - - 1. X | - - 1. X |
| - - 1. S10Ts065 | - - 1. *Tamias sibiricus* | - - 1. 2010 | - - 1. X | - - 1. X | - - 1. X |
| - - 1. S10Ts091 | - - 1. *Tamias sibiricus* | - - 1. 2010 | - - 1. 3 | - - 1. 0 | - - 1. 0 |
| - - 1. S10Ts096 | - - 1. *Tamias sibiricus* | - - 1. 2010 | - - 1. 3 | - - 1. 0 | - - 1. 0 |
| - - 1. S10Ts113 | - - 1. *Tamias sibiricus* | - - 1. 2010 | - - 1. X | - - 1. X | - - 1. X |
| - - 1. S10Ts117 | - - 1. *Tamias sibiricus* | - - 1. 2010 | - - 1. X | - - 1. X | - - 1. X |
| - - 1. S10Ts123 | - - 1. *Tamias sibiricus* | - - 1. 2010 | - - 1. X | - - 1. X | - - 1. X |
| - - 1. S10Ts125 | - - 1. *Tamias sibiricus* | - - 1. 2010 | - - 1. 0 | - - 1. 3 | - - 1. 0 |
| - - 1. S10Ts145 | - - 1. *Tamias sibiricus* | - - 1. 2010 | - - 1. 2 | - - 1. 1 | - - 1. 0 |
| - - 1. S10Ts157 | - - 1. *Tamias sibiricus* | - - 1. 2010 | - - 1. 3 | - - 1. 0 | - - 1. 1 |
| - - 1. S10Ts255 | - - 1. *Tamias sibiricus* | - - 1. 2010 | - - 1. 3 | - - 1. 0 | - - 1. 0 |
| - - 1. S10Ts309 | - - 1. *Tamias sibiricus* | - - 1. 2010 | - - 1. 3 | - - 1. 0 | - - 1. 0 |
| - - 1. S10Ts316 | - - 1. *Tamias sibiricus* | - - 1. 2010 | - - 1. 0 | - - 1. 3 | - - 1. 0 |
| - - 1. S10Ts324 | - - 1. *Tamias sibiricus* | - - 1. 2010 | - - 1. 0 | - - 1. 3 | - - 1. 0 |
| - - 1. S10Ts333 | - - 1. *Tamias sibiricus* | - - 1. 2010 | - - 1. 0 | - - 1. 3 | - - 1. 0 |
| - - 1. S06As006 | - - 1. *Apodemus sylvaticus* | - - 1. 2006 | - - 1. 3 | - - 1. 0 | - - 1. 0 |
| - - 1. S06As14 | - - 1. *Apodemus sylvaticus* | - - 1. 2006 | - - 1. 3 | - - 1. 0 | - - 1. 0 |
| - - 1. S07As020 | - - 1. *Apodemus sylvaticus* | - - 1. 2007 | - - 1. 3 | - - 1. 0 | - - 1. 0 |
| - - 1. S07As034 | - - 1. *Apodemus sylvaticus* | - - 1. 2007 | - - 1. X | - - 1. X | - - 1. X |
| - - 1. S07As048 | - - 1. *Apodemus sylvaticus* | - - 1. 2007 | - - 1. 3 | - - 1. 0 | - - 1. 0 |
| - - 1. S07As049 | - - 1. *Apodemus sylvaticus* | - - 1. 2007 | - - 1. 3 | - - 1. 0 | - - 1. 0 |
| - - 1. S07As073 | - - 1. *Apodemus sylvaticus* | - - 1. 2007 | - - 1. 3 | - - 1. 0 | - - 1. 0 |
| - - 1. S08As083 | - - 1. *Apodemus sylvaticus* | - - 1. 2008 | - - 1. X | - - 1. X | - - 1. X |
| - - 1. S08As127 | - - 1. *Apodemus sylvaticus* | - - 1. 2008 | - - 1. 3 | - - 1. 0 | - - 1. 0 |
| - - 1. S10As034 | - - 1. *Apodemus sylvaticus* | - - 1. 2010 | - - 1. X | - - 1. X | - - 1. X |
